# Energy transfer from luminescent centers to Er^3+^ in erbium-doped silicon-rich oxide films

**DOI:** 10.1186/1556-276X-8-366

**Published:** 2013-08-28

**Authors:** Lu Jin, Dongsheng Li, Luelue Xiang, Feng Wang, Deren Yang, Duanlin Que

**Affiliations:** 1 State Key Laboratory of Silicon Materials, Zhejiang University, Hangzhou 310027, People’s Republic of China; 2 Department of Materials Science and Engineering, Zhejiang University, Hangzhou 310027, People’s Republic of China

**Keywords:** Luminescence centers, Silicon nanoclusters, Erbium ion, Energy transfer, Silicon-rich oxide

## Abstract

The energy transfer mechanism between luminescent centers (LCs) and Er^3+^ in erbium-doped silicon-rich oxide (SROEr) films prepared by electron beam evaporation is investigated. Intense photoluminescence of the LCs (weak oxygen bonds, neutral oxygen vacancies, and Si=O states) within the active matrixes is obtained. Fast energy transfer from Si=O states to Er^3+^ takes advantage in the SROEr film and enhances the light emission from Er^3+^. The introduction of Si nanoclusters, which induces the Si=O states and facilitates the photon absorption of the Si=O states, is essential to obtain intense photoluminescence from both Si=O states and Er^3+^.

## Background

In the recent years, a great amount of researchers have focused on the luminescent materials of Si-based light sources to obtain efficient light emission, which is a critical step for the achievement of the Si-based optical interconnections [1]. These materials include silicon-rich oxide (SRO) [2-6], silicon-rich nitride [6,7], Ge-on-Si luminescent materials [8], and rare-earth-doped Si-based materials [9-14]. Among all these Si-based materials, erbium-doped SRO (SROEr) films have attracted a great research interest in these years as the 1.54-μm luminescence of Er^3+^ is compatible with both the optical telecommunication and the Si-based microphotonics [11-18]. The excitation mechanism of Er^3+^ in SROEr has been basically discussed, while three indirect excitation mechanisms of Er^3+^ have been proposed in the literatures: (1) slow energy transfer process (*τ*_r_ = approximately 4 to 100 μs) from exciton recombination in silicon nanoclusters (Si NCs) followed by internal relaxation to Er^3+^[11,16,18,19], (2) fast energy transfer process (nanosecond and faster) between hot carriers inside the Si NCs and Er^3+^[20,21], (3) fast energy transfer process (very fast, sub-nanosecond) from luminescent centers (LCs) in the SROEr matrixes to Er^3+^[17].

The Si NCs acting as the classical sensitizers embedded in the SROEr films can provide large excitation cross-section and efficient energy transfer to Er^3+^, from which the luminescence of Er^3+^ can be improved significantly [11]. Both light emitting diodes [12] and optical gain [13] have been achieved from the Si NC-sensitized SROEr systems. However, the luminescence intensity and optical gain of Er^3+^ are still limited due to the low fraction of Er^3+^ ions sensitized by the Si NCs [15]. Moreover, the confined carrier absorption (CCA) process that exists in the Si NC-sensitized SROEr systems would be accelerated by the slow energy transfer process between the Si NCs and Er^3+^, from which the optical properties of Er^3+^ would be further degenerated [16,17]. Besides, the introduction of nonradiative decay channels due to the presence of the Si NCs would also degenerate the optical performances of the Si NC-sensitized SROEr systems [18]. Furthermore, the luminescence intensity of Er^3+^ would be quenched by the Auger process produced during the energy transfer process between hot carriers and Er^3+^[20,21].

Compared to the indirect energy transfer process from the Si NCs and hot carriers to the nearby Er^3+^, the sensitization from the LCs in the SROEr matrixes to Er^3+^ could effectively overcome the above disadvantages, and the 1.54-μm luminescence of Er^3+^ might be improved significantly. This improvement partially originated from the “atomic”-size scale of the LCs, where the sensitizer (LCs) with high density could be obtained. Meanwhile, the CCA as well as the Auger process that existed in the Si NC-sensitized SROEr systems could be degenerated obviously since the energy transfer process from the LCs to Er^3+^ is extremely fast (*τ*_r_ = approximately 100 ns) [17]. Furthermore, the LCs could be obtained in the SROEr matrixes with low Si excesses; therefore, the nonradiative decay channels caused by the incorporation of the Si NCs could be significantly suppressed [22]. However, previous research about LC-mediated luminescence of Er^3+^ in SROEr films has shown that the LCs are unstable during the high-temperature annealing process, which limits the photoluminescence (PL) performance of both LCs and Er^3+^[17]. Therefore, intense and stable emission of LCs in SROEr film is required in the view of obtaining efficient luminescence of Er^3+^ by the energy transfer process from LCs to the Er^3+^.

In this work, SROEr films with stable LCs were prepared by electron beam evaporation (EBE) following a post-annealing process. The evolution of the PL from the SROEr films during the annealing process is investigated. The effect of energy transfer from the LCs to the nearby Er^3+^ on the luminescent performance of SROEr film is demonstrated, and the optimization of its PL property is expected. Furthermore, the effect of the introduction of Si NCs on the performance of LCs is studied.

## Methods

The SROEr films were deposited on p-type silicon substrates by EBE using a SiO and Er_2_O_3_ mixed target (Er atomic concentration of approximately 20 at%), with the deposition rate of 1 to 3 Å/s controlled by the electron beam current. The base pressure of the deposition chamber was pumped to lower than 5 × 10^−3^ Pa, and the substrates were maintained at 300°C. The atomic compositions of the as-deposited (A.D.) films were detected by Rutherford back scattering analysis using 2.02-MeV^4^ He ion beam at a scattering angle of 165°. The Si atomic concentration in the SROEr films was about 36 at%, and the Er concentration was around 3 × 10^19^ at./cm^−3^. The Er concentration was low enough to avoid the Er clustering procedure [23]. After the deposition of the SROEr films, a thermally annealing process at 700°C to 1,150°C in a quartz furnace under nitrogen ambient was experienced to form the different sensitizers (LCs and/or Si NCs). The structural characteristics of the films were studied using high-resolution transmission electron microscopy (HRTEM) image. Room temperature PL was detected by charge-coupled device (PIXIS: 100 BR, Princeton Instruments, Trenton, USA) and InGaAs photon multiple tube (PMT, Hamamatsu R5509, Iwata City, Japan) for visible and infrared emission ranges, respectively, where a He-Cd laser with a wavelength of 325 nm was employed as the excitation light source. Time-resolved PL excited by a 405-nm picosecond laser diode was performed by a multichannel photon counting system (Edinburgh Instruments Ltd., Livingston, UK). A xenon lamp with continuous wavelength in the range from 200 to 900 nm was employed for the measurement of the PL excitation (PLE) spectra. The infrared (IR) spectroscopy was performed using a Bruker IFS 66 V/S Fourier transform IR (FTIR, Bruker BioSpin AG Ltd., Beijing, China) spectroscope under the transmission mode and vacuum condition.

## Results and discussion

**Figure 1:**
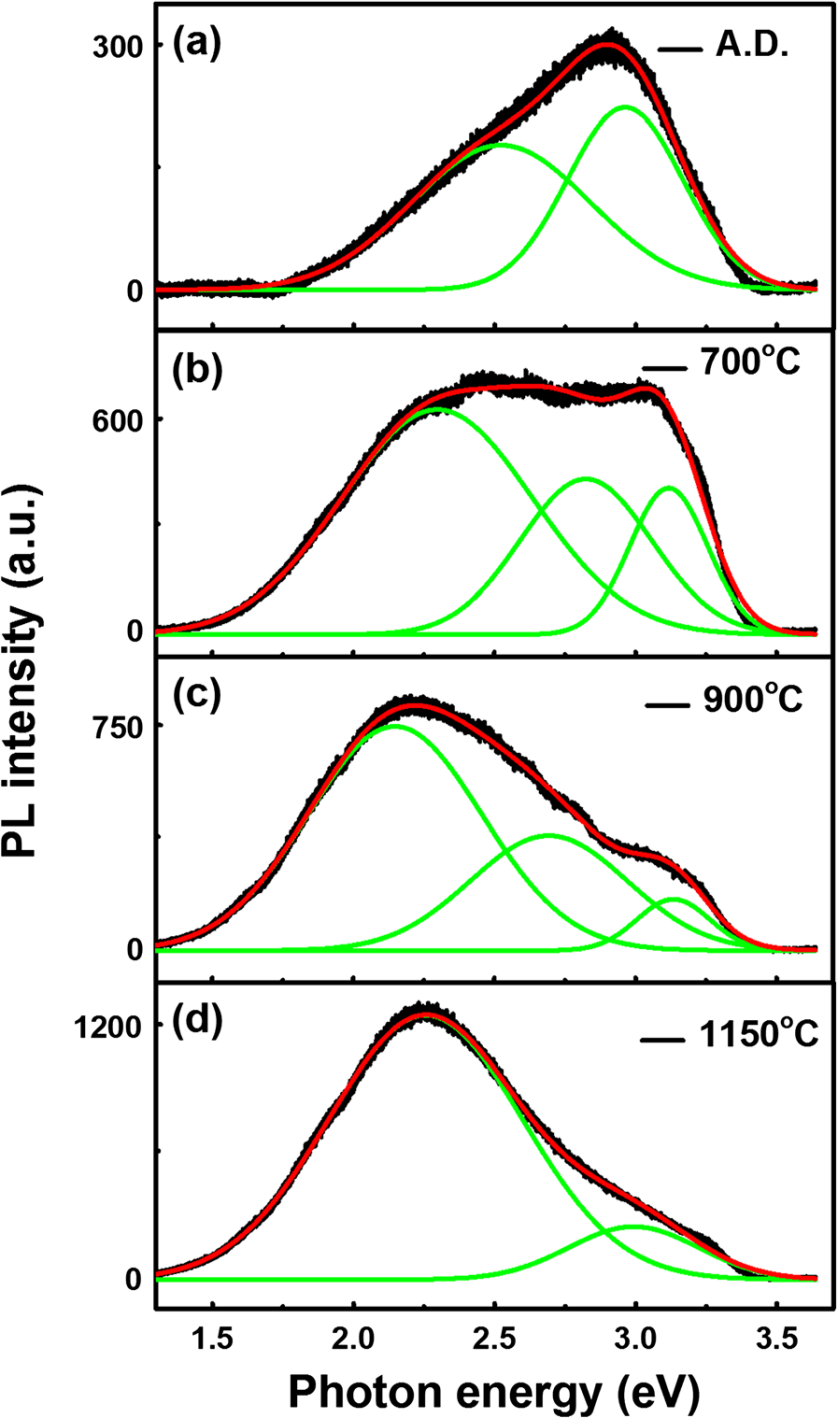
**PL spectra of SROEr films with different annealing temperatures.** PL spectra of **(a)** the A. D. SROEr film and the SROEr films annealed at **(b)** 700°C, **(c)** 900°C, and **(d)** 1,150°C in N_2_ ambience for 30 min. The experimental data is denoted by black lines, the fitting data of the general and the divided peaks are denoted by the red and green lines, respectively.

The evolution of the optical property from the SROEr matrixes with the annealing process is investigated by PL, as shown in Figure 1. From the Gauss fittings of these PL spectra, three PL bands could be resolved, which were in the ranges from 3.0 to 3.1, 2.6 to 2.8, and 2.2 to 2.5 eV, respectively. The one in the range from 3.0 to 3.1 eV originated from weak oxygen bonds (WOBs) [24], where the relative intensity of this band decreases during the annealing process. The PL band in the range from 2.6 to 2.8 eV originated from neutral oxygen vacancies (NOVs) [25]. These NOVs are instable and only exist in the annealed films with proper annealing temperatures (700°C to 900°C in our experiments). While for the dominant PL band in the range from 2.2 to 2.5 eV, either the Si NCs or the Si=O states in the matrix could contribute to it. The emission of the Si NCs could be explained by the quantum confinement model, according to which the PL band would redshift with the increasing sizes of the Si NCs [26]. However, in our experiment, the PL band in the range from 2.2 to 2.5 eV blueshifts slightly when the sizes of the Si NCs increase after high-temperature annealing (≥900°C). Hence, we consider that this PL band mainly originated from the luminescence of the Si=O states in the matrix.

**Figure 2:**
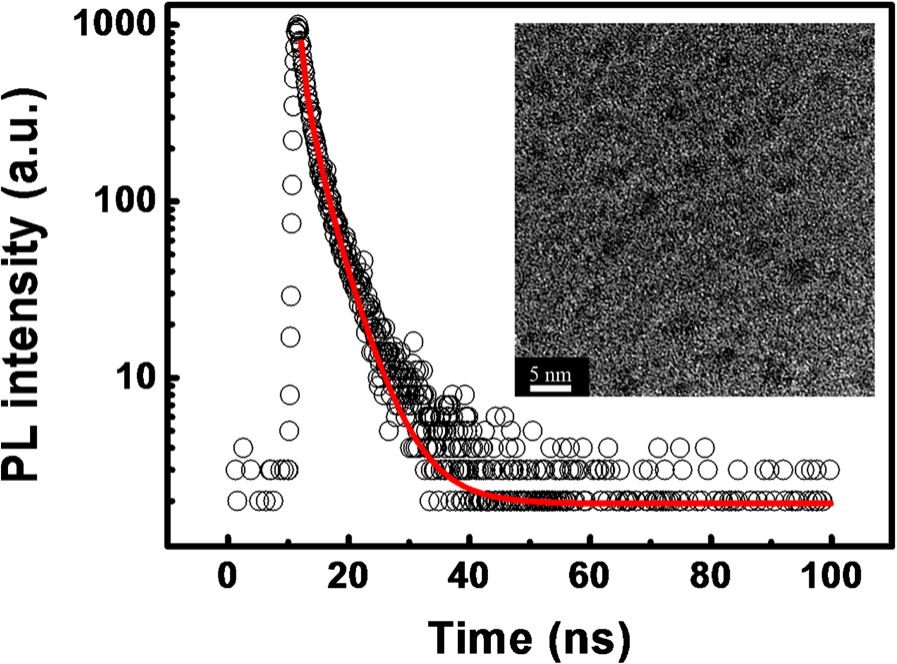
**Decay curve of PL peaked at 2.2 eV and HRTEM image for the SROEr film.** Decay curve of the PL signal recorded at 2.2 eV for the SROEr film annealed at 1,150°C for 30 min (denoted by empty circles). The experiment data is fitted by stretched exponential function (denoted by solid line). The inset shows the HRTEM image of the SROEr film annealed at 1,150°C for 30 min.

To further determine the existence and the PL mechanism of the Si NCs and the Si=O states in the matrix, the HRTEM image and the time-resolved PL spectra of the SROEr film annealed at 1,150°C for 30 min are measured, as shown in Figure 2. The high-density Si NCs with the average diameter of about 2 nm are obtained. Moreover, from the fitting of the time-resolved PL spectra by a stretched exponential function, we can obtain that the characteristic decay time of the PL peak at approximately 2.2 eV is about 1.7 ns, as shown in Figure 2, which fits well with the lifetime of the Si=O states [27]. Similar values of the characteristic decay time of this emission band (about 2.2 to 2.5 eV) could be also obtained from the as-deposited and annealed SROEr films (not shown here). Furthermore, the time-resolved PL spectrum which peaked at 2.2 eV is also detected at the time range of microsecond since the PL decay time of the Si NCs is around 100 μs [28,29]. However, the microsecond-decay dynamics is undetected in our experiments. Therefore, we attribute the luminescent band in the range from 2.2 to 2.5 eV mainly to the radiative recombination of the Si=O states in the SROEr matrix.

**Figure 3:**
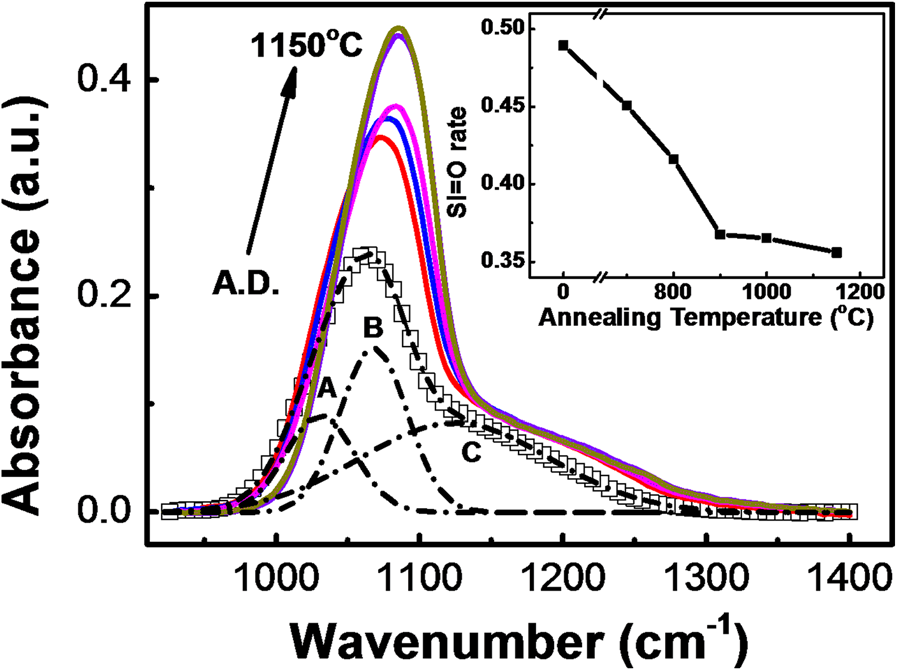
**FTIR spectra and the percentage of Si=O symmetric stretching mode for the SROEr films.** FTIR spectra of the SROEr films annealed at different temperatures in N_2_ ambience for 30 min, the FTIR spectra of the A.D. sample is denoted by empty square and that of the annealed samples are denoted by the colored lines (red, 700°C; blue, 800°C; magenta, 900°C; violet, 1,000°C; and dark yellow, 1,150°C). A typical fitting of the FTIR spectra is provided for the A.D. sample (the fitting data is denoted by dash dot line). The sub-peaks A, B, and C represent the components from the Si-O-Si bulk, Si-O-Si surface, and Si=O symmetric stretching modes, respectively. The inset shows the percentage of the Si=O symmetric stretching mode for the SROEr films with different annealing temperatures.

The FTIR spectra of the SROEr films with various annealing temperatures confirm the impact of the Si=O states on the luminescent band in the range from 2.2 to 2.5 eV, as shown in Figure 3. The intensity of the main peak (1,065 to 1,085 cm^−1^) characterized by the Si-O-Si stretching mode [30] enhances gradually with the increase of the annealing temperatures. Meanwhile, the position of this peak is redshifted to a higher wavenumber, which indicates the phase decomposition of the SROEr matrix (see our previous paper in [4]). Moreover, three Gaussian bands could be resolved, as shown in Figure 3, which represent the Si-O-Si bulk stretching mode (sub-peak A), Si-O-Si surface stretching mode (sub-peak B), and Si=O symmetric stretching mode (sub-peak C) [16]. Interestingly, the rate of the Si=O symmetric stretching mode in the SROEr films gradually decreased with the increase of the annealing temperatures, as shown in the inset of Figure 3, which is opposite to our previous investigations on SRO matrixes without the doping of Er [6]. This decrease might be caused by the activation of the Er ions in the SROEr matrixes to their trivalent coordination [31], where the Si=O bonds would be decomposed significantly. Importantly, the downtrend of the percentage of the Si=O symmetry slows down obviously for the SROEr films annealed above 900°C, as shown in the inset of Figure 3, illustrating the serious clustering of the Si NCs that induce the Si=O states. Moreover, the introduction of the Si NCs would also facilitate photon absorption of the Si=O states. It is worth to note that enhanced PL intensity of the Si=O states has been obtained after high-temperature annealing despite the reduction of the concentration of the Si=O states, as shown in Figure 1. This might be caused by the introduction of the Si NCs in the SROEr matrix after high-temperature annealing, from which the energy transfer between the Si NCs and the Si=O states would enhance the PL intensity of the Si=O states.

**Figure 4:**
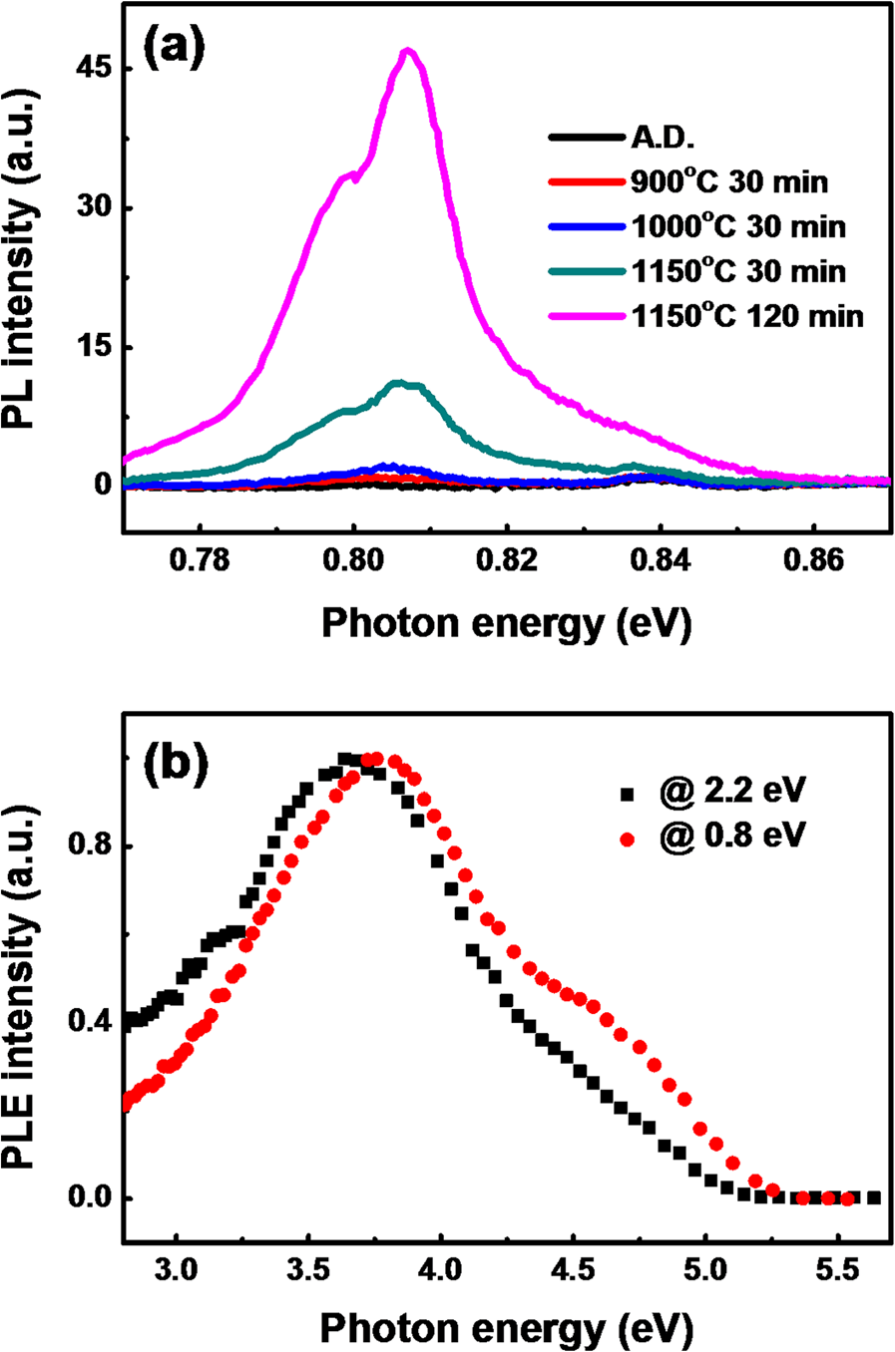
**PL spectra of Er**^**3+ **^**ion and PLE spectra of both Er**^**3+ **^**ion and Si=O states. (a)** PL spectra of the Er^3+^ ions in the SROEr films with various annealing conditions. **(b)** Normalized PLE spectra of the Si=O states (collected at 2.2 eV) and Er^3+^ (collected at 0.8 eV) for the SROEr film annealed at 1,150°C for 30 min.

Obviously, the LCs (WOBs, NOVs, Si=O states, and so on) could act as the sensitizers in the SROEr matrixes. For the investigation of the energy transfer from these sensitizers to Er^3+^, the PL spectra of Er^3+^ in the infrared band (^4^I_15/2_ to ^4^I_13/2_) were measured, as shown in Figure 4a. Interestingly, the PL signal from Er^3+^ could not be detected from the SROEr films annealed at <900°C, although the intense visible PL from the LCs (WOBs, NOVs, and Si=O states) can be observed. However, for the samples annealed above 900°C, the PL of Er^3+^ could be obviously resolved (its intensity increases significantly with the annealing temperatures). Therefore, the energy transfer from the NOVs could be excluded since the NOVs disappear after high-temperature annealing (1,150°C). Moreover, the sensitization of the temperature-dependent PL of Er^3+^ from the WOBs could also be excluded due to their almost identical PL from the as-deposited and annealed SROEr films. Meanwhile, the evolution of the PL intensity from Er^3+^ is in accordance with that from the Si=O states at higher-annealing temperatures (≥900°C, the critical temperature that the Si NCs begin to precipitate in a great amount). Hence, we consider that the sensitization of Er^3+^ is mainly caused by the Si=O states in the SROEr matrix. According to the discussion above, the Si=O states would be induced greatly when the Si NCs precipitate in a great amount, and the energy transfer process between the Si=O states and Er^3+^ is also controlled by the Si NCs in the SROEr matrix. The introduction of the Si NCs can not only enhance the luminescence of the Si=O states by facilitating the photon absorption of the Si=O states but also improve the PL of Er^3+^ by the energy transfer process of the Si=O states. Besides, the PL of Er^3+^ would also be enhanced by the activation of Er^3+^ in the SROEr films after high-temperature annealing (≥900°C). The PL intensity of Er^3+^ increased significantly when the annealing time increased from 30 to 120 min for the SROEr annealed at 1,150°C, as shown in Figure 4a. It means that further improvement of the PL property of Er^3+^ could be achieved by optimizing the annealing condition of the SROEr films.

To further determine the energy transfer mechanism in the SROEr films, the PLE spectra of the Si=O states (collected at 2.2 eV) and Er^3+^ (collected at 0.8 eV) for the SROEr film annealed at 1,150°C for 30 min were measured, as shown in Figure 4b, with the intensities normalized by their correspondingly maximal values. The well overlap between the PLE spectrum for the Si=O states and that for Er^3+^ indicates that energy transfer from the Si=O states to Er^3+^ plays a dominant role in this SROEr film. The little discrepancy between these two spectra might have originated from the resonant excitation of Er^3+^. Besides, the peak around 3.8 eV which appears in the PLE spectra might be related to the optical excitation of the Si NCs since the introduction of the Si NCs would enhance the PL intensity of both Si=O states and Er^3+^.

## Conclusions

In summary, the efficient luminecence of Er^3+^ in the SROEr film is achieved by the energy transfer process from fast recombination centers (LCs). The SROEr films with abundant LCs (WOBs, NOVs, and Si=O states) and Si NCs are prepared by electron beam evaporation following a post-annealing process. Intense and stable PL of LCs dominated by the Si=O states is obtained in the SROEr matrix. From the investigation of the evolution of the PL properties and microstructures from the SROEr films, we consider the fast energy transfer from the Si=O states to Er^3+^ as the main transfer mechanism. The introduction of the Si NCs induces the Si=O states and facilitates the photon absorption of the Si=O states, which is essential to obtain intense PL from both Si=O states and Er^3+^. Further improvement of the PL property of both the Si=O states and Er^3+^ might be achieved by optimizing the annealing condition of the SROEr films.

## Abbreviations

CCA: Confined carrier absorption; EBE: Electron beam evaporation; FTIR: Fourier transform infrared spectroscope; HRTEM: High-resolution transmission electron microscopy; LCs: Luminescent centers; NOVs: Neutral oxygen vacancies; PL: Photoluminescence; PLE: PL excitation; Si NCs: Silicon nanoclusters; SROEr: Erbium-doped silicon-rich oxide; WOBs: Weak oxygen bonds.

## Competing interests

The authors declare that they have no competing interests.

## Authors’ contributions

LJ performed the experiments, collected and analyzed the data, and wrote the paper. DL conceived the experiments, analyzed the results, and wrote the paper. LX, FW, DY, and DQ helped with the data analysis and wrote the paper. All authors read and approved the final manuscript.

## Authors’ information

DL received his Ph.D. degree in the State Key Laboratory of Silicon Materials and Department of Material Science and Engineering from Zhejiang University, Hangzhou, China, in 2002. He is currently an Associate Professor in the Department of Material Science and Engineering at Zhejiang University. His current research interests include the synthesis of plasmonic microstructure, application of plasmonic microstructure on solar cells, Raman and luminescence, and silicon photonics. LJ, LX, and FW are currently Ph.D. students in the State Key Laboratory of Silicon Materials and Department of Materials Science and Engineering, Zhejiang University, Hangzhou, China. Their current research interests include luminescence from erbium-doped silicon-rich oxide matrix, silicon-rich nitride matrix, and dislocations in silicon, silicon nitride-based light-emitting devices, and localized surface plasmon resonance of metal nanostructures. DY received his B.S. degree from Zhejiang University, Hangzhou, China, in 1985, and Ph.D. degree in Semiconductor Materials from the State Key Laboratory of Silicon Materials in Zhejiang University, Hangzhou, China, in 1991. He has been with the Institute of Metal Materials in Tohoku University, Japan, and worked for Freiberg University, Germany, from 1995 to 1997. He is currently the director of the State Key Laboratory of Silicon Materials. His current research interests include the fabrication of single crystalline silicon materials for ultra-larger-scale integrated circuit and defect engineering, polysilicon materials and compound thin film photo-electric conversion materials for photovoltaic, nano-scale silicon wire/tube and other one-dimensional semiconductor materials, and silicon-based materials for optoelectronics. DQ received his B.S. degree from the Department of Electrical Engineering from Xiamen University, Xiamen, China, in 1951. He has been with the Department of Electrical Engineering, Department of Radio-Based Semiconductor Materials and Devices, Department of Materials Science and Engineering in Zhejiang University, China, since 1953.

## References

[B1] Paniccia M Morse M Salib M Integrated photonics Top Appl Phys 2004 8 51 88 10.1007/978-3-540-39913-1_2

[B2] Cheng CH Lien YC Wu CL Lin GR Multicolor electroluminescent Si quantum dots embedded in SiO_*x*_ thin film MOSLED with 2.4% external quantum efficiency Opt Express 2013 8 391 403 10.1364/OE.21.000391 23388932

[B3] Pavesi L Negro LD Mazzoleni C Franzò GF Priolo F Optical gain in silicon nanocrystals Nature 2000 8 440 444 10.1038/35044012 11100719

[B4] Jin L Li D Yang D Que D Modulation effect of microstructures in silicon-rich oxide matrix on photoluminescence from silicon nanoclusters prepared by different fabrication techniques Appl Phys A 2012 10.1007/s00339-012-7496-z

[B5] Lin G-R Lin C-J Lin C-K Chou L-J Chueh Y-C Oxygen defect and Si nanocrystal dependent white-light and near-infrared electroluminescence of Si-implanted and plasma-enhanced chemical-vapor deposition-grown Si-rich SiO_2_ J Appl Phys 2005 8 094306 10.1063/1.1886274

[B6] Lin G-R Pai Y-H Lin C-T Chen C-C Comparison on the electroluminescence of Si-rich SiN_*x*_ and SiO_*x*_ based light-emitting diodes Appl Phys Lett 2010 8 263514 10.1063/1.3459144

[B7] Wang F Li D Yang D Que D Enhancement of orange-yellow electroluminescence extraction from SiN_x_ light-emitting devices by silver nanostructures Opt Express 2013 8 846 854 10.1364/OE.21.000846 23388978

[B8] Liu J Sun X Kimerling LC Michel J Direct-gap optical gain of Ge on Si at room temperature Opt Lett 2009 8 1738 1740 10.1364/OL.34.001738 19488166

[B9] Li D Zhang X Jin L Yang D Structure and luminescence evolution of annealed Europium-doped silicon oxides films Opt Express 2010 8 27191 27196 10.1364/OE.18.027191 21196996

[B10] Jin L Li D Xiang L Wang F Yang D Que D The modulation on luminescence of Er^3+^-doped silicon-rich oxide films by the structure evolution of silicon nanoclusters Nanoscale Res Lett 2013 8 34 10.1186/1556-276X-8-34 23331713 PMC3563518

[B11] Kik PG Brongersma ML Polman A Strong exciton-erbium coupling in Si nanocrystal-doped SiO_2_ Appl Phys Lett 2000 8 2325 10.1063/1.126334

[B12] Iacona F Pacifici D Irrera A Miritello M Franzò G Priolo F Sanfilippo D Di Stefano G Fallica PG Electroluminescence at 1.54 μm in Er-doped Si nanocluster-based devices Appl Phys Lett 2002 8 3242 10.1063/1.1516235

[B13] Han HS Seo SY Shin JH Optical gain at 1.54 μm in erbium-doped silicon nanocluster sensitized Waveguide Appl Phys Lett 2001 8 4568 4570 10.1063/1.1419035

[B14] Lin G-R Lin C-J Chen C-Y Enhanced pumping energy transfer between Si nanocrystals and erbium ions in Si-rich SiO_*x*_ sputtered using Si/Er_2_O_3_ encapsulated SiO Substrate J Nanosc Nanotechnol 2007 8 2847 2851 10.1166/jnn.2007.867 17685306

[B15] Wojdak M Klik M Forcales M Gusev OB Gregorkiewicz T Pacifici D Franzò G Priolo F Iacona F Sensitization of Er luminescence by Si nanoclusters Phys Rev B 2004 8 233315

[B16] Kik PG Polman A Gain limiting processes in Er-doped Si nanocrystal waveguides in SiO_2_ J Appl Phys 2002 8 534 10.1063/1.1418417

[B17] Savchyn O Ruhge FR Kik PG Todi RM Coffey KR Nukala H Heinrich H Luminescence-center-mediated excitation as the dominant Er sensitization mechanism in Er-doped silicon-rich SiO_2_ films Phys Rev B 2007 8 195419

[B18] Pacifici D Franzò G Priolo F Iacona F Negro LD Modeling and perspectives of the Si nanocrystals–Er interaction for optical amplification Phys Rev B 2003 8 245301

[B19] Watanabe K Fujii M Hayashi S Resonant excitation of Er^3+^ by the energy transfer from Si nanocrystals J Appl Phys 2001 8 4761 4767 10.1063/1.1409572

[B20] Izeddin I Moskalenko AS Yassievich IN Fujii M Gregorkiewicz T Nanosecond dynamics of the near-infrared photoluminescence of Er-Doped SiO_2_ sensitized with Si Nanocrystals Phys Rev Lett 2006 8 207401 17155714 10.1103/PhysRevLett.97.207401

[B21] Izeddin I Timmerman D Gregorkiewicz T Moskalenko AS Prokofiev AA Yassievich IN Energy transfer in Er-doped SiO_2_ sensitized with Si nanocrystals Phys Rev B 2008 8 035327 10.1103/PhysRevLett.97.207401 17155714

[B22] Kanjilal A Rebohle L Voelskow M Skorupa W Helm M Gain limiting processes in Er-doped Si nanocrystal waveguides in SiO_2_ J Appl Phys 2008 8 103522 10.1063/1.3021414

[B23] Prtljaga N Navarro-Urrios D Tengattini A Anopchenko A Ramírez JM Rebled JM Estradé S Colonna JP Fedeli JM Garrido B Pavesi L Limit to the erbium ions emission in silicon-rich oxide films by erbium ion clustering Opt Mater Express 2012 8 1278 1285 10.1364/OME.2.001278

[B24] Cheang-Wong JC Oliver A Roiz J Hernanaez JM Rodriguez-Fernandez L Morales JG Crespo-Sosa A Optical properties of Ir^2+^-implanted silica glass Nucl Instrum Methods Phys Res B 2001 8 490 494

[B25] Song HZ Bao XM Li NS Zhang JY Relation between electroluminescence and photoluminescence of Si^+^-implanted SiO_2_ J Appl Phys 1997 8 4028 4032 10.1063/1.365712

[B26] Cho EC Green MA Xia J Corkish R Reece P Gal M Clear quantum-confined luminescence from crystalline silicon/SiO_2_ single quantum wells Appl Phys Lett 2004 8 2286 10.1063/1.1691489

[B27] Brewer A von Haeftena K *In situ* passivation and blue luminescence of silicon clusters using a cluster beam/H_2_O codeposition production method Appl Phys Lett 2009 8 261102 10.1063/1.3167355

[B28] Grom GF Lockwood DJ McCaffrey JP Labbé HJ Fauchet PM White B Jr Diener J Kovalev D Koch F Tsybeskov L Ordering and self-organization in nanocrystalline silicon Nature 2000 8 358 361 10.1038/35030062 11014187

[B29] Delley B Steigmeier EF Tamm states in finite semiconductor superlattices: influence of accumulation and depletion layers Phys Rev B 1993 8 1397 1382 10.1103/physrevb.47.1379 10006148

[B30] Martínez JR Ruiz F Vorobiev FYV Pérez-Robles F González-Hernández J Infrared spectroscopy analysis of the local atomic structure in silica prepared by sol–gel J Chem Phys 1998 8 7511 7514 10.1063/1.477374

[B31] Adler DL Jacobson DC Eaglesham DJ Marcus MA Benton JL Poate JM Citrin PH Local structure of 1.54‒μm‒luminescence Er^3+^ implanted in Si Appl Phys Lett 1992 8 2181 2183 10.1063/1.108288

